# Diversity and composition of plant species in the forest over limestone of Rajah Sikatuna Protected Landscape, Bohol, Philippines

**DOI:** 10.3897/BDJ.8.e55790

**Published:** 2020-12-29

**Authors:** Wilbert A. Aureo, Tomas D. Reyes, Francis Carlo U. Mutia, Reizl P. Jose, Mary Beth Sarnowski

**Affiliations:** 1 Department of Forestry and Environmental Sciences, College of Agriculture and Natural Resources, Bohol Island State University, Bohol, Philippines Department of Forestry and Environmental Sciences, College of Agriculture and Natural Resources, Bohol Island State University Bohol Philippines; 2 Central Visayas Biodiversity Assessment and Conservation Program, Research and Development Office, Bohol Island State University, Bohol, Philippines Central Visayas Biodiversity Assessment and Conservation Program, Research and Development Office, Bohol Island State University Bohol Philippines; 3 Institute of Renewable Natural Resources, College of Forestry and Natural Resources, University of the Philippines Los Baños, Laguna, Philippines Institute of Renewable Natural Resources, College of Forestry and Natural Resources, University of the Philippines Los Baños Laguna Philippines; 4 United States Peace Corps Philippines, Diosdado Macapagal Blvd, Pasay, 1300, Metro Manila, Philippines United States Peace Corps Philippines, Diosdado Macapagal Blvd, Pasay, 1300 Metro Manila Philippines

**Keywords:** Central Visayas, endemic, plant habit, species diversity, threatened species

## Abstract

Rajah Sikatuna Protected Landscape (RSPL), considered the last frontier within the Central Visayas region, is an ideal location for flora and fauna research due to its rich biodiversity. This recent study was conducted to determine the plant species composition and diversity and to select priority areas for conservation to update management strategy. A field survey was carried out in fifteen (15) 20 m x 100 m nested plots established randomly in the forest over limestone of RSPL from July to October 2019. Three hundred and sixty eight (368) species of plants were identified up to species level. This represented 327 angiosperms, one gymnosperm and 40 pteridophytes. Common plant families with more than 10 representative species were Moraceae, Meliaceae, Lauraceae, Dipterocarpaceae, Rubiaceae, Myrtaceae, Phyllanthaceae, Annonaceae, Araceae and Lauraceae. There were 93 (28%) endemic and 46 (14%) threatened species (vulnerable to critically endangered) observed. The cluster analysis and species accumulation curve suggests that plant species are not homogeneously distributed which implies that different management and conservation strategies should be implemented across RSPL. These results not only indicate the importance of RSPL, but also highlights areas with higher diversity and concentration of threatened and endemic species as a special area of concern. Furthermore, areas with high biodiversity value were recommended for immediate protection, while areas with low biodiversity value were recommended for reforestation programmes using species with high importance value.

## Introduction

The Philippines is one of the mega diverse (Mittermeier et al. 1997) and “hot spot” countries, with more than 10,107 described plants (Barcelona et al. 2013) and supporting 1.9% of the world’s endemic plant and vertebrate species (Myers et al. 2000). Over 57% of the major faunal and floral groups in the Philippines occur nowhere else in the world (Oliver and Heaney 1996). Bohol Island, in particular, has significant remaining forested areas within the 93,000 hectares in Rajah Sikatuna Protected Landscape (RSPL), the Loboc Watershed Reserve (LWR) and Alejawan-Cansuhay-Anibongan River Watershed Forest Reserve. Previous studies reported many indigenous timbers, including 16 species of dipterocarps (Fernando et al. 2009), 20 premium trees, one conifer, 35 edible fruit-bearing native trees, 169 ferns and fern allies (Barcelona et al. 2006) and the rest are lesser-used species in Bohol Province (Reyes Jr et al. 2015). Despite these few studies conducted, Bohol exibits a high number of recorded plant species.

The RSPL is one of the last parts in Bohol with relatively-undisturbed forest with a high diversity of plant species (Vegchel 2013). In the past, huge quantities of hardwood species, such as red lauan, *Shorea negrosensis* and white lauan, *Shorea contorta*, have been harvested. RSPL is on the southern part of the Island and all of it is mainly on karst limestone (Fernando et al. 2009). Unlike the very scientific and organised conservation programmes being poured into other forest ecosystems in the country, there is no consensus effort that would ensure effective biodiversity conservation and forest rehabilitation where a forest over limestone ecosystems and diverse fragmented forest remains with an imminent threat. Furthermore, despite being declared a protected landscape, its remaining forests have declined to only 4% from their original full-forested area due to anthropogenic and climatic pressures (Bullecer and Socorin 2013).

RSPL was declared a national park in 1987 and a protected landscape in 2000 by the Department of Environment and Natural Resources (DENR) (Ecosystem Research and Development Bureau, DENR 2005). Soil Water Conservation Foundation (SWCF), a non-government organisation, pioneered conservation assessment in 1996, which became the basis for the said declaration. However, SWCF has stopped biodiversity assessment and monitoring activities, with the Bohol Environment Management Office (BEMO) now taking point.

Meanwhile, there is growing emphasis on the importance of conservation planning which identifies priority areas and directs limited conservation resources in a strategic manner to help address challenges to biological diversity. Approaches in conservation planning have evolved over the past few decades from focusing mainly on species to encompassing broader aspects of biodiversity. Gordon et al. 2005 identified six criteria relating to biodiversity value (e.g. the number of endemic species, conservation status, diversity, variety of taxa) to select priority areas for conservation. Thus, the present study aimed to select priority areas for conservation in RSPL using six biological parameters, namely: diversity, richness, abundance, basal area and number of threatened and endemic species.

## Materials and Methods

### Study site

**Figure 1.**
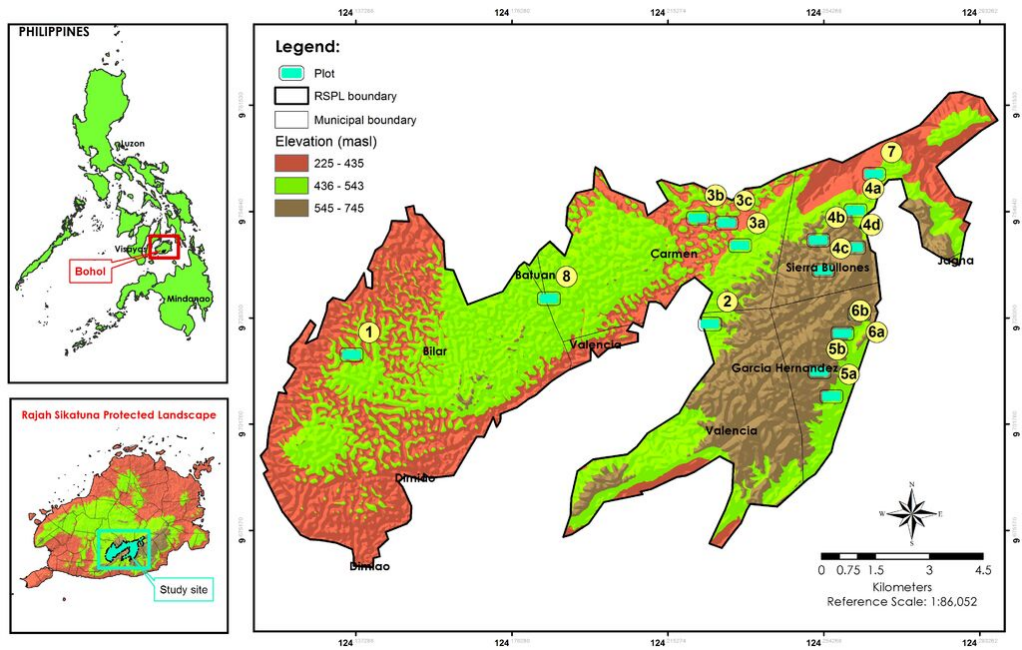
Elevation map of Rajah Sikatuna Protected Landscape with the locations of the sampling plots.

Rajah Sikatuna Protected Landscape is located in the six municipalities of Batuan, Bilar, Carmen, Dimiao, Garcia Hernandez, Sierra Bullones and Valencia (Fig. 1), with a total land area of 10,452.6 hectares. RSPL is about 8 km inland from the national road of Bilar, in the low mountain range in the south of Bohol Island. Practically all of the remaining contiguous forest in Bohol is inside this park, with only patches of intermingled plantations and dipterocarp forest elsewhere on the island. RSPL is characterised by rolling hills with remnant natural forest on steep limestone terrain, surrounded by plantation forest, deforested hills and grassland. Limestone molave forest covers 60% of the landscape, grassland 15%, forestry and agro-industrial plantations 5% and permanent agricultural areas 10%. The protected area is a popular site for birdwatchers, with many recent records of threatened and restricted-range species of the Mindanao and Eastern Visayas Endemic Bird Areas. The floral habitats within RSPL support six species of large mammals; Philippine tarsier (*Carlito syrichta*), long-tailed macaque (*Macaca fascicularis*), Philippine flying lemur (*Cynocephalus volans*), Malay civet (*Viverra tangalunga*), common palm civet (*Paradoxurus hermaphroditus*) and wild pig (*Sus philippinensis*) (Mallari et al. 2001). Table 1 shows the sampling location and the description of the plots which were randomly selected using ArcGIS software and were established in each sampling site.

### Field Data Collection

This study was conducted from 22 July- 7 October 2019. We followed the random nested sampling plot design by Lillo et al. 2019 for a better understanding of the stand structure of the forest from the ground to the canopy. Fifteen (15) sampling plots, each with a dimension of 20 m x 100 m were used. Each sampling plot was divided into five (5) equal segments (20 m x 20 m) to facilitate recording of canopy species with diameter at breast height (DBH) of 10 cm and above. Nested subplots of 5 m x 5 m were then laid at the centre of each segment for data recording of plants in the intermediate layer having DBH of less than 10 cm. Further, four small nested plots (1 m x 1 m) were used to record all plants less than 1 m tall including understorey plants, ground cover and seedlings.

Data recorded in the field were: (i) plant names from family down to species level; (ii) diameter at breast height (cm) and total height (m) of species in the canopy layer; (iii) plant groups of observed plants; and (iv) GPS coordinates of all corners of each segment and nested plots. Relative locations of trees were sketched for tracing and monitoring purposes. For small-sized plants (understorey and ground vegetation), these data were obtained; (i) number of individuals and (ii) estimated percent crown cover.

Identification and nomenclature were aided using the following strategies: (i) expert determination; (ii) use of flora databases (Co's Digital Flora of the Philippines 2011; Plant of the World Online 2017; International Plant Name Index (IPNI) 2016; Flora Malesiana 2019), (iii) lexicons (Salvosa 1963, Rojo 2011), (iv) published books (Flora Malesiana, Flora de Manila, Enumeration of Flowering Plant), field guides and other literature (e.g. De Guzman et al. 1986, Rojo and Aragones 1997, Fernando et al. 2004, Reyes 2007, Lapitan et al. 2010, McPherson and Amoroso 2011, Amoroso 2013, Tandang et al. 2014 and Malabrigo et al. 2016); and finally (v) use of digital images.

### Data Analyses

**Importance value**

The relative density, relative frequency and relative dominance for each tree species in all plots were determined to obtain their importance value (IV), a standard measure in ecology that determines the rank relationships of species. A high importance value of species indicates a composite score for high relative species dominance, density and frequency and provides a basis on what species can be used for restoration.

To compute for the relative density, relative dominance and relative frequency, the following formulae were used (Mueller-Dombois and Ellenberg 1974).

*Density = total number of individuals of a species / area sampled*

*Relative Density = density of species / total densities of all species x 100*

*Dominance = basal area (DBH area) of species / total area sampled*

*Relative Dominance = dominance of species / total dominance of all species x 100*

*Occurrence = number of times a species is encountered / total number of plots established*

*Frequency = number of occurrences / total number of occurrences*

*Relative Frequency = frequency of species / total of frequencies x 100*

*Importance value (IV) = Relative Density + Relative Dominance + Relative Frequency*

**Diversity index**

Shannon-Weiner (H') and Simpson (D) indices and eveness were determined using the equations of Magurran (1998). These were calculated using the following formulae:

*Shannon Diversity Index (H') = -∑ pi(LNpi)*

where pi is the proportion (n/N) of individuals of one particular species found (n) divided by the total number of individuals found (N)

*Evenness Index (E') = H’/LN(s)*

where s = number of species

*Simpson Diversity Index (D) = 1 - (∑n(n-1)/N(N-1)*

where n = the total number of organisms of a particular species; N = the total number of organisms of all species

The description of the diversity index value is presented in Table 2

**Cluster analaysis and species accumulation curve**

The species accumulation curve (SAC) was done using *specaccum* (vegan) method = random and fitted values, residuals, non-linear model coefficients using the *fitspecaccum* and *hclust()* functions in R package version 3.6.3 (R Core Team 2013). We used "random" because we want to determine the mean and standard deviation of SAC from random orders of the data or without subsample replacement (Gotelli and Colwell 2001). Additionally, this method can take weights that give the sampling effort for each site. Cluster analysis of plots was done using the Bray-Curtis Similarity Index from Paleontological Statistics (PAST version 2.17c) (Hammer and Harper 2006). The dendogram was generated through the unweighted pair-group method (UPGMA) and bootstrapping (n = 1000). We employed this method of analysis because it is sensitive to small sample sizes and missing observations.

### Conservation status and endemicity

The conservation status of plant species was based on local, ‘‘The National List of Threatened Philippine Plants and their Categories” (DENR 2017) and international, the IUCN Red List (IUCN 2019) catergorisations. Endemicity was determined through a Philippine archive of plant species (Co's Digital Flora of the Philippines 2011) which is available online.

### Selecting priority areas

We identified six criteria which relate to biodiversity value (e.g. computed diversity, richness, abundance, importance value, basal area and number of threatened and endemic species) to select priority areas for conservation. Some of these criteria (e.g. endemicity, conservation status, richness) were used by Gordon et al. 2005 and Replan and Malaki 2017. We added diversity and abundance to further explain the variability of plant species and basal area to indicate large, adult trees which could serve as good sources of seeds/wildlings, as well as significant stocks of carbon. Plots were ranked using the values of each criterion. Since there were 15 plots, the highest value in each criterion was weighted with value 15 while the lowest had 1. The sum of all weighted points in each criterion was summed-up and ranked to determine the priority areas.

## Results

**Floristic composition**

Rajah Sikatuna Protected Landscape was recorded to have 368 plant species belonging to 223 genera from 101 families. Angiosperms were the most diverse represented by 327 species, while there were 40 species of pteridophytes and a single gymnosperm species (*Gnetom gnemon*) (Table 3). At family level, the most abundant taxa included Moraceae, Meliaceae, Lauraceae, Dipterocarpaceae, Rubiaceae, Myrtaceae, Phyllantaceae, Annonaceae, Araceae and Lauraceae. The remaining 91 plant families were represented by less than 10 species. With regards to plant genera, the richest recorded were: *Aglaia*, *Asplenium*, *Canarium*, *Ficus*, *Hopea*, *Neonauclea*, *Shorea* and *Syzgium*.

**Figure 2.**
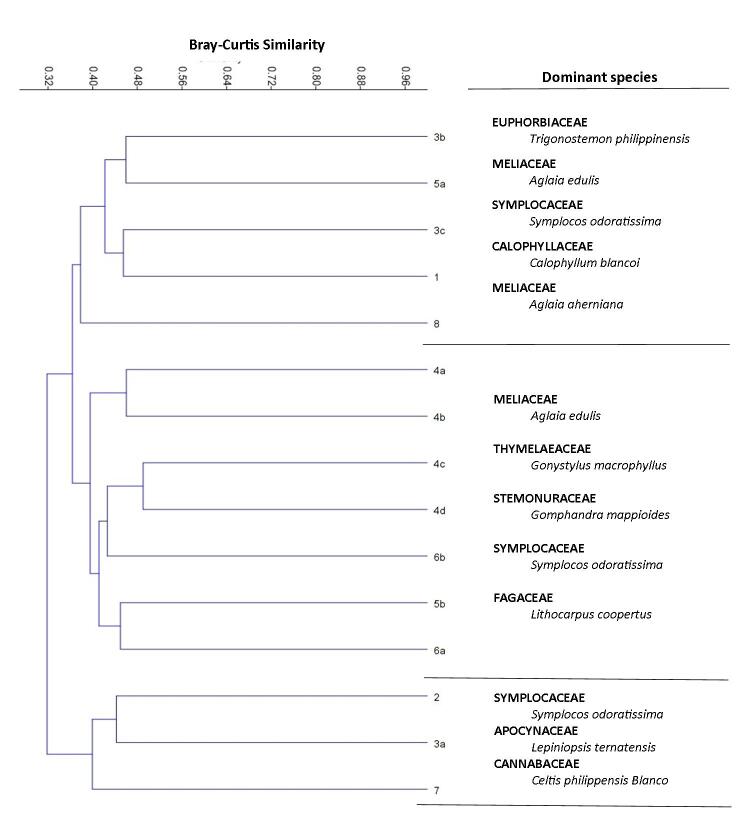
Dendogram of 15 sampling plots generated through UPGMA using the Bray-Curtis Similarity Index. Bootstraping was done at n = 1000; cophenetic correlation is 0.75.

**Figure 3.**
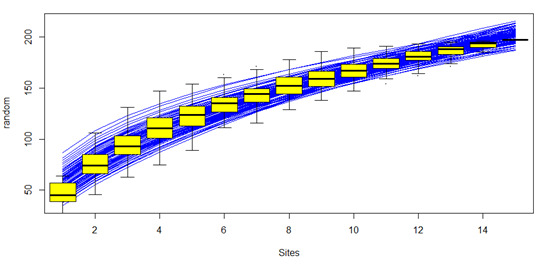
Species accumulation curve (SAC) of recorded plant species in each plot.

In the analysis of similarity of vegetation structure and composition of each plot, three main floristic groups were identified (Fig. 2). Cluster 1 had five plots grouped together, in which four of them were characterised by severe incision, dominated by *Trigonostemon philippinensis*. On the other hand, cluster 2, grouped by seven plots, was characterised by higher plant composition compared to the other clusters. Moreover, plant species were characterised and dominated by *Diplodiscus paniculatus*, *Phaeanthus ophthalmicus*, *Shorea squamata*, *Lithocarpus coopertus*, *Gomphandra mappioides*, *Donax canniformis* and *Shorea contorta*. Lastly, three plots were grouped to form cluster 3, in which all of them were characterised by severe incision, all dominated by *Symplocos odoratissima* and *Lepiniopsis ternatensis*. Furthermore, our SAC (Fig. 3) implies that more species were discovered with increasing number of sampling plots.

**Plant diversity**

**Figure 4.**
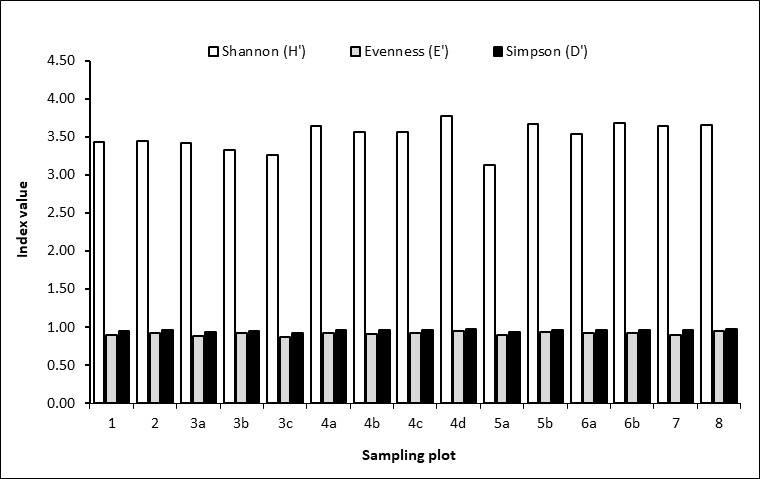
Species diversity indices (Shannon and Simpson) in each plot.

High diversity was observed in all established plots in RSPL (Fig. 4). All plots have more than 90 species and 240 individuals (Table 7). Nan-od had the highest value of the Shannon index (H' = 3.77), while Cambuyo had the lowest (H' = 3.13). In terms of species, Datag had the highest number with value 134, followed by Nan-od with 133, then the lowest was Montehermoso with 93. Meanwhile, the individuals in Datag had the highest with a value of 337, followed by Nan-od with 332 and lastly Cabacnitan with 248.

**Species importance value**

Based on the computed IV (Table 4), *Trigonostemon philippinensis* had the highest importance value in plots 1 and 5a. Meanwhile, *Symplocos odoratissima* had the highest importance value in plots 2, 3a and 7. The highest computed importance value of 93.16 for *Symplocos odoratissima* was observed in plot 3a. Moreover, dipterocarp species, such as *Shorea contorta* and *Shorea squamata*, had the highest importance value in plots 6b, 4a and 4c, respectively.

**Tree stucture and density**

**Figure 5.**
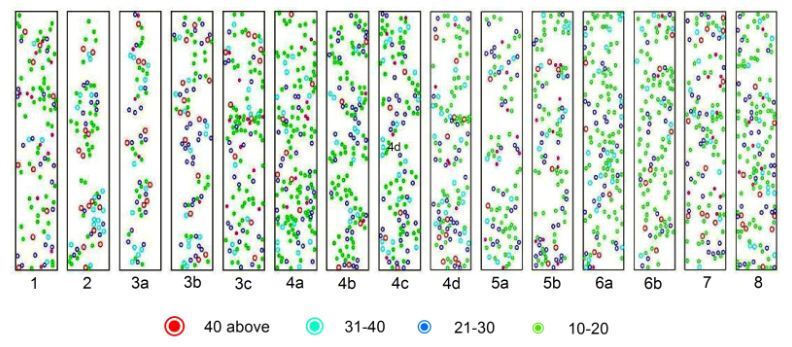
Structure and density of tree species recorded in each plot of Rajah Sikatuna Protected Landscape. Diameter class per plot is reflected in centimetres.

Tree species with diameter class (DBH) of 10-20 cm had the highest proportion of individuals across all size classes (57%), followed by 21-30 cm (24%), 31-40 cm (11%) and 40+ cm (7%) (Table 5). High percentage of 10-20 cm and 21-30 cm diameter classes were observed in plot 3b (Monthermoso), while 31-40 cm was in plot 4a (Nan-od) and 40+ cm was in plot 2 (Omjon). Moreover, the computed basal area for 10-20 cm, 21-30 cm, 31-40 cm and 40+ cm diameter classes were 1.76, 0.89, 0.37 and 0.35 m^2^/ha which totalled 12.05 m^2^/ha in all plots. A trend on decreasing number of trees with increasing diameter class was also observed (Fig. 5).

**Threatened and endemic species**

**Figure 6.**
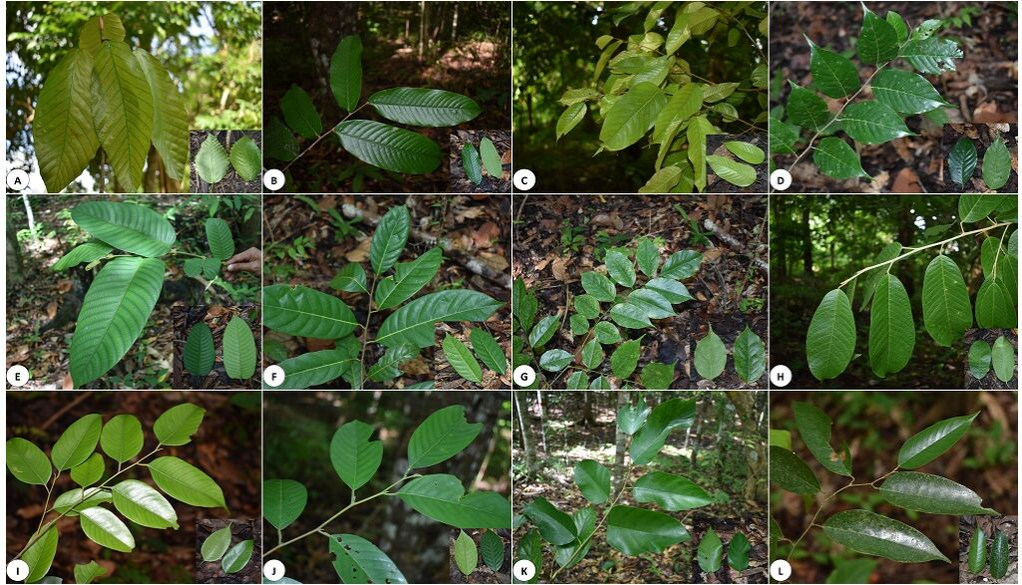
Some threatened tree species of Rajah Sikatuna Protected Landscape. *Dipterocarpus grandiflorus* (**A**), *Hopea philippinensis* (**B**), *Shorea guiso* (**C**), *Hopea acuminata* (**D**), *Shorea squamata* (**E**), *Vatica mangachapoi* (**F**), *Pterocarpus indicus* (**G**), *Anisoptera thurifera* (**H**), *Hopea quisumbingiana* (**I**), *Shorea polysperma* (**J**), *Shorea contorta* (**K**) and *Shorea astylosa* (**L**).

Forty-six (46) species were recorded as threatened (critically endangered, endangered and vulnerable) under either the Philippine Red List (DENR 2017) or the IUCN (2019) (Table 6). Ninety three Philippine endemic species were recorded, of which 37 species were accounted in the plot established in Nan-od. The lowest was 22 accounted in the plot established in Bilar. Of these 46 threatened species, 13 were critically endangered, nine were endangered and 24 were vulnerable. The highest number of threatened species (37) was recorded in the plot established in Nan-od, followed closely by the plots in Datag and Omjon, both with 36 species. The plot with the least number of threatened species of 20 was the plot in Cambuyo. Noteworthy threatened species were White lauan (*Shorea contorta*) (Fig. 5K), Mayapis (*Shorea squamata*) (Fig. 5D) and Manggachapui (*Hopea acuminata*) (Fig. 6E) which are listed as critically-endangered species, but were found to be quite common in the area. Other critically-endangered species, including *Dipterocarpus grandiflorus* (Fig. 5A), *Hopea philippinensis* (Fig. 5B), *Shorea guiso* (Fig. 5C), *Vatica mangachapoi* (Fig. 5F), *Pterocarpus indicus* (Fig. 5G), *Anisoptera thurifera* (Fig. 5H), *Hopea quisumbingiana* (Fig. 5I), *Shorea polysperma* (Fig. 5J) and *Shorea astylosa* (Fig. 5L), were also observed in the area.

### Biodiversity value

The biodiversity value of an area is always measured in terms of species richness and the number of endemic and threatened species present (Replan and Malaki 2017). These criteria were used for the selection of priority areas for conservation with the addition of diversity, abundance and total basal area. Results showed that Barangays Datag, Nan-od, Bugsoc and Omjon were amongst the areas which ranked the highest (Table 7). On the other hand, Barangays Cambuyo, Montehermoso and Bilar ranked the lowest.

## Discussion

The study provides baseline data on plant composition in the forest over limestone of the Rajah Sikatuna Protected Landscape. A total of 368 plant species, belonging to 223 genera from 101 families, were recorded. This result is higher compared to similar studies conducted in forest over limestone of Dinagat Island which accounted for 144 plant species, belonging to 50 families and 88 genera (Lillo et al. 2018) and 192 species with159 genera belonging to 62 plant families from Canbantug forest in Cebu (Replan and Malaki 2017). Aside from the family Dipterocarpaceae, we observed similar dominant plant families of Moraceae, Meliaceae, Lauraceae, Rubiaceae, Myrtaceae, Phyllantaceae, Annonaceae, Araceae and Lauraceae with the latter studies. Species belonging to the family Dipterocarpaceae, which were commonly found in this study, are known as good sources of timber and are considered economically important. It is worth noting that most of the dipterocarp species reported by Fernando et al. 2009 were recorded in this study, with the addition of *Shorea negrosensis*, *Shorea astylosa*, *Shorea polysperma* and *Hopea foxworthyi*. The observation of these four dipterocarp species is attributed to the expanded sampling area which was not surveyed by the previous study. On the other hand, *Shorea malibato* and *Shorea polita* were not observed in this particular study because these species are considered rare and are mostly found in non-limestone forest of Bohol (Fernando et al. 2009). Meanwhile, the number of pteridophyte species (40 species) recorded in this study was comparably lower than the number in Barcelona et al. 2006 of 169 species. This may be due to the higher sampling effort and larger area (18 localities) covered compared to the eight localities surveyed in this study. Results of SAC and the cluster analysis showed that species are not homogeneously distributed which implies that different management and conservation strategies should be implemented across the RSPL.

Species were also observed to form their own phytosociological group, distinct from each other. *Symplocos odoratissima*, in particular, was found common on top of the mountain hills though was also present in relatively flat terrain. *Trigonostemon philippinensis* and *Pterocymbium tinctorium*, on the other hand, formed thickets along valleys and flat rocky substrates in between mountain hills which may occasionally be submerged in flowing water during rainy season, while *Shorea contorta* and *Shorea squamata* extended their dominance from hilly areas to relatively rolling undisturbed closed canopy forests. These observations conformed to the findings of Reyes Jr et al. 2015.

All plots showed high diversity in both Shannon (H’) and Simpson’s (D) Diversity Indices, with the plot in Brgy. Nan-od exhibited the highest value of 3.77, followed by Plot 6b in Datag, while the lowest diversity was recorded in Plot 5a in Cambuyo. It is important to note that, in terms of the Shannon Diversity Index, the ordering of the plots was likely affected by the topography, dense forest cover and maturity of the forest (Replan and Malaki 2017) where the plots were established. Barangays, Nan-od and Datag are relatively-young secondary forests characterised by smaller-sized and stunted trees as evidenced by the high basal area of diameter class (10-20). In addition, the topography in these areas is relatively flat to rolling. Jiao-jun et al. 2007 stated that increasing level of disturbance in secondary forest increases the number of species which likely explains the high diversity in these areas. On the other hand, Montehermoso and Omjon are evidently older second-growth forests comprised of few larger trees and less understorey species with sloping topography.

Of the 368 plant species encountered, 93 species were found endemic to the Philippines and 46 were listed as threatened, based on IUCN 2019 and DENR 2017. Of these endemic species, 91 were flowering seed plants and two species were pteridophytes. The highest number (37) of endemic species was recorded in Nan-od while the highest number (21) of threatened species was recorded in Omjon. The number of endemic species found in this study (93) is higher compared to the 23 species recorded by Lillo et al. 2018) in Dinagat Island and 19 recorded by Replan and Malaki 2017) in Canbantug forest, Cebu. In terms of threatened species, the number recorded here (46) is higher than the 18 species in Canbantug forest (Replan and Malaki 2017) and the 25 species in Dinagat Island (Lillo et al. 2018).

### Priority areas for conservation

Areas with the highest totals, Datag, Nan-od, Bugsoc and Omjon, summed from the identified criteria, need the most immediate conservation actions. The species in these areas are highly threatened due to their economic value, being locally accessible and often used for construction and as firewood. Barangays Bugsoc, Datag and Nan-od exhibited very high species diversity, especially in the canopy layer (tree species). These Barangays are similarly the areas displaying high basal area of tree species mostly belonging to the family Dipterocarpaceae - a very good source of timber - highlighting the risk of future exploitation. In contrast, Barangays Montehermoso, Bilar and Cambuyo exhibit relatively-lower species diversity. These areas are located near communities where we can expect high human encroachment in the forest. In addition, it is worth mentioning that despite low diversity in Barangay Montehermoso, *Shorea negrosensis*, a critically-endangered species was only observed in this area, thereby presenting a unique conservation priority. While this present study does not account for all species within RSPL, these results provide a data-driven basis for prioritisation of areas for conservation.

## Conclusions and Recommendations

RSPL has a very high diversity value and is a habitat to at least 368 flora species. A total of 93 plants are exclusively found in the Philippines and a significant number of forty six (46) threatened species were observed in the area. Many of these threatened species like White lauan (*Shorea contorta*), Mayapis (*Shorea squamata*) and Mangachapui (*Hopea acuminata*), although listed as critically endangered, were commonly found within its natural forest. Therefore, it is recommended that immediate conservation and management activities should be conducted to parts of RSPL with high diversity and number of threatened and endemic species like Barangays Bugsoc, Datag, Nan-od and Omjon to save these plants from extirpation. However, for areas with low diversity and number of threatened and endemic plants, we suggest that reforestation initiatives, like assisted natural regeneration (ANR) and rain-forestation, be implemented. Additionally, species with high importance value in each area are also recommended to be used in the said reforestation programme. Lastly, future studies should also consider plant associations and environment interactions.

## Supplementary Material

3CFACE9A-EC90-50AE-974B-8EE106239F59 10.3897/BDJ.8.e55790.suppl1 Supplementary material 1 List of plant species in Rajah Sikatuna Protected Landscape Data type Occurrences of plants (Excel) Brief description This contains all the raw data and analyses performed in this article File: oo_438468.xlsx https://binary.pensoft.net/file/438468 Wilbert A. Aureo, Francis Carlo U. Mutia

## Figures and Tables

**Table 1.** The general and specific locality of sampling sites in Rajah Sikatuna Protected Landscape (RSPL) with the plot codes (Plot) used in the hierarchical cluster analysis. Geographic coordinates (Lat: latitude, Long: longitude) in each plot are in decimal format.

**Plot**	**Municipality**	**Barangay**	**Total land area (ha)**	**Forest cover (%)**	**Topography**	**Lat / Long**
1	Bilar	Zamora	459.87	80	Steep to rolling	9.7192, 124.1362
2	Valencia	Omjon	714.63	60	Sloping	9.7268, 124.2257
3a	Carmen	Montehermoso	350.69	50	Sloping	9.7460, 124.2330
3b	Carmen	Montehermoso			Rolling	9.7531, 124.2234
3c	Carmen	Montehermoso			Sloping	9.7527, 124.2300
4a	Sierra Bullones	Nan-od	812.91	85	Relatively flat to rolling	9.7520, 124.2618
4b	Sierra Bullones	Nan-od			Relatively flat	9.7472, 124.2573
4c	Sierra Bullones	Nan-od			Relatively flat	9.7490, 124.2627
4d	Sierra Bullones	Nan-od			Flat to rolling	9.7452, 124.2560
5a	Garcia Hernandez	Cambuyo	403.12	65	Rolling	9.7088, 124.2561
5b	Garcia Hernandez	Cambuyo			Rolling	9.7094, 124.2543
6a	Garcia Hernandez	Datag	252.55	75	Flat to rolling	9.7291, 124.2641
6b	Garcia Hernandez	Datag			Flat to rolling	9.7262, 124.2645
7	Sierra Bullones	Bugsoc	190.56	50	Steep to rolling	9.7631, 124.2693
8	Batuan	Cabacnitan	306.46	50	Relatively flat	9.7229, 124.1867

**Table 2.** Classification of diversity indices interpreted using the descriptions proposed by Fernando 1998).

**Relative Value Rating**	**Species Diversity (H’)**	**Evenness (E’)**
Very High	3.50– above	0.75–1.00
High	3.00–3.49	0.50–0.74
Moderate	2.50–2.99	0.25–0.49
Low	2.00–2.49	0.15–0.24
Very Low	0.00–1.99	0.05–0.14

**Table 3.** Plant groups of the species in each family recorded in RSPL. The entire list of plant species is available in Suppl. material 1.

**Family**	**Angiosperm**	**Gymnosperm**	**Pteridophyte**	**Total species**
Acanthaceae	2			2
Aceraceae	1			1
Actinidiaceae	1			1
Anacardiaceae	6			6
Annonaceae	12			12
Apocynaceae	8			8
Araceae	11			11
Araliaceae	5			5
Arecaceae	7			7
Asparagaceae	1			1
Aspleniaceae			7	7
Asteraceae	1			1
Athyriaceae			1	1
Begoniaceae	1			1
Bignonaceae	1			1
Blechnaceae			1	1
Boraginaceae	2			2
Brownlowiaceae	1			1
Burseraceae	7			7
Calophyllaceae	2			2
Cannabaceae	1			1
Casuarinaceae	1			1
Celastraceae	4			4
Clusiaceae	2			2
Combretaceae	2			2
Connaraceae	1			1
Cornaceae	3			3
Cunoniaceae	1			1
Davalliaceae			1	1
Dilleniaceae	1			1
Dipterocarpaceae	17			17
Dryopteridaceae			2	2
Ebenaceae	3			3
Elaeocarpaceae	2			2
Euphorbiaceae	10			10
Fabaceae	15			15
Fagaceae	1			1
Flagellariaceae	1			1
Gesneriaceae	2			2
Gnetaceae		1		1
Hymenophyllaceae			3	3
Hypericaceae	2			2
Hypoxidaceae	1			1
Juglandaceae	1			1
Lamiaceae	2			2
Lauraceae	10			10
Lecythidaceae	1			1
Leeaceae	1			1
Lindsaeaceae			1	1
Lomariopsidaceae			2	2
Lygodiaceae			1	1
Magnoliaceae	3			3
Malvaceae	1			1
Maranthaceae	1			1
Marattiaceae			1	1
Meliaceae	17			17
Menispermaceae	5			5
Monimiaceae	1			1
Moraceae	21			21
Myristicaceae	3			3
Myrsinaceae	1			1
Myrtaceae	13			13
Nephrolepidaceae			3	3
Nyctaginaceae	1			1
Oleaceae	1			1
Orchidaceae	3			3
Pandanaceae	2			2
Pentapetaceae	2			2
Pentaphylaceae	1			1
Phyllanthaceae	15			15
Piperaceae	2			2
Pittosporaceae	1			1
Poaceae	2			2
Polygalaceae	1			1
Polypodiaceae			3	3
Primulaceae	2			2
Proteaceae	1			1
Pteridaceae			2	2
Rhamnaceae	1			1
Rosaceae	3			3
Rubiaceae	18			18
Rutaceae	7			7
Salicaceae	2			2
Sapindaceae	6			6
Sapotaceae	8			8
Schizaeaceae			1	1
Selaginellaceae			4	4
Simaroubaceae	1			1
Smilacaceae	1			1
Sparmanniaceae	1			1
Stemonuraceae	1			1
Sterculiaceae	5			5
Strombosiaceae	1			1
Symplocaceae	2			2
Tectariaceae			4	4
Thelypteridaceae			3	3
Thymelaeaceae	4			4
Urticaceae	7			7
Vitaceae	3			3
Zingiberaceae	2			2
**Total**	**327**	**1**	**40**	**368**

**Table 4.** Highest percent importance value (IV) of species in each plot in RSPL.

**Plot**	**Latin name**	**Family name**	**Common name**	**IV**
1	*Trigonostemon philippinensis* Stapf in Elmer	Euphorbiaceae	Croton	44.01
	*Mallotus cumingii* Müll.Arg.	Euphorbiaceae	Apanang	16.02
	*Shorea contorta* S.Vidal,	Dipterocarpaceae	White lauan	15.84
2	*Symplocos odoratissima* (Blume)	Symplocaceae	Bayokbok	33.02
	*Hopea acuminata* Merr.	Dipterocarpaceae	Manggachapui	31.41
	*Tarennoidea wallichii* (Hook.f.)	Rubiaceae	Pototan gubat	21.83
3a	*Symplocos odoratissima* (Blume)	Symplocaceae	Bayokbok	93.16
	*Diplodiscus paniculatus* Turcs.	Brownlowiaceae	Balobo	49.36
	*Aglaia exstipulata* (Griff.) W.Theob.	Meliaceae	Aglia gagmay	15.12
3b	*Chisocheton cumingianus* (C.DC.)	Meliaceae	Balukanag	38.70
	*Pterocymbium tinctorium* (Blanco) Merr	Sterculiaceae	Taluto	37.93
	*Trigonostemon philippinensis* Stapf in Elmer	Euphorbiaceae	Croton	37.48
3c	*Ficus ampelas* Burm.f.	Moraceae	Upling gubat	31.37
	*Trigonostemon philippinensis* Stapf in Elmer	Euphorbiaceae	Croton	28.49
	*Chisocheton cumingianus* (C.DC.)	Meliaceae	Balukanag	17.04
4a	*Shorea squamata* (Turcz.) Benth. & Hook.f. ex DC	Dipterocarpaceae	Mayapis	34.31
	*Symplocos odoratissima* (Blume)	Symplocaceae	Bayokbok	22.66
	*Diplodiscus paniculatus* Turcs.	Brownlowiaceae	Balobo	20.18
4b	*Alangium meyeri* Merr.	Cornaceae	Putian	56.36
	*Pterospermum celebicum* Miq.	Pentapetaceae	Bayokbayokan	11.14
	*Litsea cordata* (Jack) Hook.f.	Lauraceae	Marang timber	10.20
4c	*Shorea squamata* (Turcz.) Benth. & Hook.f. ex DC	Dipterocarpaceae	Mayapis	34.13
	*Pterocymbium tinctorium* (Blanco) Merr	Sterculiaceae	Taluto	27.27
	*Chisocheton cumingianus* (C.DC.)	Meliaceae	Balukanag	21.25
4d	*Lithocarpus coopertus* (Blanco)	Fagaceae	Ulaian	26.56
	*Shorea squamata* (Turcz.) Benth. & Hook.f. ex DC	Dipterocarpaceae	Mayapis	24.37
	*Hopea acuminata* Merr.	Dipterocarpaceae	Manggachapui	19.46
5a	*Trigonostemon philippinensis* Stapf in Elmer	Euphorbiaceae	Croton	32.70
	*Aglaia edulis* (Roxb.) Wall.	Meliaceae	Malasaging	29.19
	*Chisocheton cumingianus* (C.DC.)	Meliaceae	Balukanag	22.88
5b	*Gomphandra mappioides* Valeton, Crit. Overz	Stemonuraceae	Taguibokbok	32.84
	*Chisocheton cumingianus* (C.DC.)	Meliaceae	Balukanag	24.57
	*Hopea acuminata* Merr.	Dipterocarpaceae	Manggachapui	17.71
6a	*Pterocymbium tinctorium* (Blanco) Merr	Sterculiaceae	Taluto	43.46
	*Symplocos odoratissima* (Blume)	Symplocaceae	Bayokbok	23.02
	*Lithocarpus coopertus* (Blanco)	Fagaceae	Ulaian	20.07
6b	*Shorea contorta* S.Vidal,	Dipterocarpaceae	White lauan	34.20
	*Trigonostemon philippinensis* Stapf in Elmer	Euphorbiaceae	Croton	18.09
	*Baccaurea lanceolata* (Miq.)	Phyllanthaceae	Mala Ulalian	14.62
7	*Symplocos odoratissima* (Blume)	Symplocaceae	Bayokbok	38.35
	*Lepiniopsis ternatensis* Valeton	Apocynaceae	Paginga	19.80
8	*Lithocarpus coopertus* (Blanco)	Fagaceae	Ulaian	15.89
	*Trigonostemon philippinensis* Stapf in Elmer	Euphorbiaceae	Croton	15.59
	*Shorea guiso* (Blanco) Blume	Dipterocarpaceae	Guijo	15.49

**Table 5.** Diameter classes and total basal area of tree species in RSPL.

**Diameter class** (cm)	**Plot**																
	**1**	**2**	**3a**	**3b**	**3c**	**4a**	**4b**	**4c**	**4d**	**5a**	**5b**	**6a**	**6b**	**7**	**8**	**Total**	%
10 to 20	69	35	23	31	76	102	83	64	61	66	60	86	93	95	69	1013	57
21 to 30	20	18	20	33	24	30	42	38	33	31	26	22	29	32	31	429	24
31 to 40	5	16	13	13	12	15	11	21	20	11	10	9	19	14	11	200	11
41 above	11	13	8	14	10	7	6	5	11	5	2	8	4	10	12	126	7

**Table 6.** Conservation status and endemicity of plant species recorded in each plot.

**Conservation status**	**Plot**														
	**1**	**2**	**3a**	**3b**	**3c**	**4a**	**4b**	**4c**	**4d**	**5a**	**5b**	**6a**	**6b**	**7**	**8**
Threatened	25	36	21	19	20	26	23	33	37	20	27	23	36	29	27
Endemic	22	27	28	24	29	24	34	31	37	30	27	29	29	25	31

**Table 7.** Priority areas for conservation ranked using biodiversity values.

**Plot**	**Endemic**	**Richness**	**Abundance**	**Threatened**	**Basal area**	**Diversity**	**Rank**
6b	29	134	331	36	145	3.68	1
4d	37	123	325	37	125	3.77	2
4b	34	133	332	23	142	3.56	3
4c	31	109	307	33	128	3.56	4
6a	29	124	337	23	125	3.54	5
7	25	101	325	29	151	3.64	6
4a	24	95	272	26	154	3.64	7
2	27	105	326	36	82	3.45	8
8	31	101	248	27	123	3.65	9
5b	27	102	268	27	98	3.67	10
1	22	107	317	25	105	3.43	11
3a	28	114	307	21	64	3.42	12
3c	29	100	285	20	122	3.26	13
5a	30	100	302	20	113	3.13	14
3b	24	93	255	19	91	3.33	15

## Hosting institution

Bohol Island State University (BISU)
